# Invited reply to the letter to the editor by McNally et al., 2021

**DOI:** 10.1186/s12891-021-04118-7

**Published:** 2021-03-08

**Authors:** Christian Morgenstern, Nora Renz, Sabrina Cabric, Elena Maiolo, Carsten Perka, Andrej Trampuz

**Affiliations:** Center for Musculoskeletal Surgery (CMSC), Charité – Universitätsmedizin Berlin, Corporate member of Freie Universität Berlin, Humboldt-Universität zu Berlin and Berlin Institute of Health, Charitéplatz 1, 10117 Berlin, Germany

Thank you for the opportunity to respond to the comments on our paper Morgenstern et al. 2020 [1]. The detection of microbial heat production (microcalorimetry) has been studied for several biological and medical applications and has shown superior sensitivity and/or shorter time to microbial detection compared to conventional culture [2–4]. In our study we aimed to evaluate the performance of microcalorimetric analysis of synovial fluid in comparison to synovial fluid culture for the preoperative diagnosis of periprosthetic joint infection (PJI).

In their Letter to the Editor (McNally et al. 2021), the authors raised their concern over the use of unvalidated definition criteria of PJI, which could potentially lead to overdiagnosing of infection. It is correct that the definition criteria we used for this study are highly sensitive and could be prone to over-diagnosing rather than underdiagnosing infection. Still, this classification system is presently being used in the clinical routine in many institutions (including ours) and has also been employed in several other studies [5–16].

In the last years, various PJI diagnostic criteria have been proposed and used by different authors. In the time our prospective study was conducted (2014 to 2015), the International Consensus Meeting (ICM) criteria [17], the Musculoskeletal Infection Society (MSIS) criteria [18] and the Infectious Disease Society of America (IDSA) criteria [19] were the most popular criteria available for PJI definition. However, we believed that these criteria were not appropriate for our study as they present crucial drawbacks. For instance, IDSA criteria do not consider an important criterion for PJI, namely the synovial fluid leukocyte count, which is inappropriate for a study that focuses on the preoperative investigation of synovial fluid. The 2013 ICM criteria were mainly based on expert opinion and studies with a low quality of evidence [20]. Furthermore, sonication of removed implants, which represents the most sensitive intraoperative method to detect low-virulent pathogens [21], was not considered neither in ICM nor in IDSA criteria, which consequently made them miss a considerable number of PJI [9, 10]. In fact, in a cohort of 136 patients with a painful prosthetic joint, Huard et al. [10] reported 41 PJI applying MSIS criteria, 50 with IDSA and 68 with proposed European Bone and Joint Infection Society (EBJIS) (our institutional) criteria, confirming the results published by Renz et al. [9]. ICM criteria predominantly missed those infections caused by low-virulent pathogens [9]. Another study showed that presence of a single minor criterion (but not fulfilling the 2013 ICM criteria for PJI) was associated with worse outcome of total joint revision [22]. These observations resulted in the modification of the ICM PJI definition criteria in 2018 [23].

In regard to the other comments, we are not ignoring the value of preoperative synovial biopsies as claimed by McNally et al. Still, we believe that this invasive procedure (requiring arthroscopy or open biopsy) should be performed only exceptionally, i.e. in case of a dry tap or inconclusive arthrocentesis results, as it poses intervention-related risk of additional infection. In addition, arthroscopically collected samples are probably not most representative for diagnosis of low-grade infection, as the biofilm location at the prosthesis-bone interface cannot be easily reached. The additional risk of performing a biopsy needs to be weighed against the moderate gain of additional information compared to only performing arthrocentesis. Some institutions still perform open biopsy as part of diagnostic routine, which, in our opinion, should be done only in exceptional cases with a good rational indication.

The optimal cutoff value for synovial fluid leukocyte count that allows discriminating chronic PJI (occurring after > 4 weeks postoperatively or with a symptom duration of > 4 weeks) from aseptic failure has been suggested by different investigators ranging from 1100/μl to 4200/μl. When considering studies reporting a realistic rate of low-virulent causing pathogens (e.g. coagulase-negative staphylococci), referred cutoff values were below 2000/μl [24, 25]. Regarding the histopathology [26], the result of 1 to 10 granulocytes per high-power field was used for histopathological diagnosis of PJI. The cutoff of 2 granulocytes per high-power field in this study based on the recent report of Krenn et al. [27] demonstrating a cut-off of 23 granulocytes in a total of 10 high-power fields as optimal threshold, which proportionally translates to 2 granulocytes per high-power field (averaged over 10 fields).

Based on these facts, we firmly disagree with McNally et al.’s assertion that our study could be misleading or biased. The definition criteria in our study were delineated in the Methods section and individually listed in the Results section, which allows for an adequate interpretation of the results by the reader. We agree that by using highly sensitive criteria to define infection, the performance of all evaluated diagnostic tests could be underestimated. However, as the performance of all diagnostic tests was assessed using the same definition criteria, the alleged “bias” would therefore equally apply to all the evaluated diagnostic methods without a selective disadvantage to a particular method. In fact, the positivity rate of synovial fluid culture and microcalorimetry in the present study was equal (39 and 39%, respectively), as it was in a previous study investigating culture and microcalorimetry of synovial fluid of native joint for the diagnosis of infection (46 and 46%, respectively) [3].

Most recently, the PJI definition criteria of the EBJIS have been published by most of the authors that redacted the Letter we are herewith replying to [28]. Our institutional criteria were presented by one author of our group (AT) during the 36th Annual Meeting of EBJIS in 2017, and thereafter have been referred to in many studies as “proposed EBJIS criteria” [5–10, 12–16, 28, 29]). The meeting recommended a reappraisal of the definition categories which resulted in the publication of the official EBJIS criteria in 2021 [28]. In fact, in the recently published paper by EBJIS [28], the described confirmative definition criteria represent modified versions of our institutional definition criteria.

We believe that the new EBJIS definition criteria are robust and superior to previously used criteria (MSIS, ICM, IDSA). We regret that our previous contribution in the development of the PJI definition criteria was not appropriately credited in the recent publication of McNally et al. [28] by indicating the original publications.

In conclusion, PJI definition criteria are a matter of continuous debate and there is not one set of criteria that has been universally accepted or validated. Despite some investigators claim to use validated PJI definition criteria [23, 29, 30], none of these criteria were appropriately validated on an independent large cohort with long-term follow-up. Considering patient conditions and expectations, microbiology, intervention-related risks, ethical considerations and functional outcome, the treating physician needs to decide individually whether to use highly sensitive criteria (and thereby overdiagnose infection with a risk of overtreatment) or highly specific criteria (and thereby miss some infections, potentially resulting in subsequent treatment failure with all related consequences). In many institutions (including ours), highly sensitive definition criteria are used as clinical decision support tool. We must accept that it is not always possible to establish a clear diagnosis of infection and aseptic failure, with a remaining “gray zone” of inconclusive cases. Therefore, each case must be individually evaluated before deciding on the most appropriate therapy, independently from the definition criteria employed.

We hope that this response will contribute to the discussions on finding the best PJI definition criteria in the future. For full transparency, raw data of this study are provided as Supplementary Material.

Yours sincerely,

Christian Morgenstern.

Nora Renz.

Sabrina Cabric.

Elena Maiolo.

Carsten Perka.

Andrej Trampuz.

## Supplementary Information

**Additional file 1.**

## Data Availability

For full transparency, raw data of this study are provided as Supplementary Material.

## Abbreviations

EBJIS: European Bone and Joint Infection Society
IDSA: Infectious Disease Society of America
ICM: International Consensus Meeting
MSIS: Musculoskeletal Infection Society
PJI: Periprosthetic joint infection
